# Extracellular vesicles-miR-205-5p inhibits lymphatic metastasis in pancreatic cancer through diffusely downregulating VEGFA

**DOI:** 10.7150/jca.110659

**Published:** 2025-03-29

**Authors:** Yuanyang Wang, Cheng Qin, Yutong Zhao, Bangbo Zhao, Zeru Li, Tianyu Li, Xiangyu Zhang, Weibin Wang

**Affiliations:** Department of General Surgery, State Key Laboratory of Complex Severe and Rare Diseases, Peking Union Medical College Hospital, Chinese Academy of Medical Sciences and Peking Union Medical College, Beijing, 100730, China.

**Keywords:** Pancreatic cancer, Extracellular Vesicles, miR-205-5p, lymph node metastasis, VEGFA.

## Abstract

Pancreatic ductal adenocarcinoma (PDAC) is to become the second leading cause of cancer-related death by 2040. Many factors contribute to this dilemma, including lymphatic metastasis, which is the primary cause of PDAC metastasis. The inhibition of early lymph node metastasis, including the lymphangiogenic process, may be a novel strategy for PDAC treatment. Through miRNA sequencing of plasma extracellular vesicles (EVs) from PDAC patients, for the first time, we identified that plasma EV-miR-205-5p served as a non-invasive biomarker distinguishing lymphatic metastasis status (N0 vs. N2) in PDAC patients. Using tissue microarray and *in situ* hybridization, we discovered that miR-205-5p was highly expressed in PDAC, but negatively correlated with lymph node metastasis. By *in vivo* and *in vitro* experiments, we demonstrated its unique mechanism of action via EV-mediated transfer to human lymphatic endothelial cells (HLECs), leading to systematic downregulation of VEGFA and inhibition of the Akt/Erk pathway, which suppressed lymphangiogenesis. Delivering miR-205-5p via engineered EVs might be a promising strategy to eliminate PDAC lymphatic metastasis and improve prognosis.

## Introduction

Pancreatic ductal adenocarcinoma (PDAC) has become a significant public health threat to human life and health, with a trend of becoming the second leading cause of cancer-related deaths by 2040, surpassing colorectal cancer 1. A combination of multiple factors contributes to the lethality of PDAC, including difficulty in detection before the progression stage and its high metastatic potential 2, 3. Lymph node (LN) metastasis is the primary pathway of PDAC metastasis that requires lymphangiogenesis, which serves as the first step in the formation and expansion of lymphatic vessels from the existing lymphatic systems 4. Inhibition of early lymph node metastasis, including the lymphangiogenic process, is crucial for limiting distant metastasis of PDAC.

Extracellular vesicles (EVs) refer to small membrane vesicles (30-150 nm) that contain DNA, RNA, proteins, and lipids and might regulate multiple biological functions 5, 6. Tumor cell-derived EVs and their contents may contribute to tumor diagnosis as well as the development of new therapeutic targets and anticancer drug carriers 7. As reported, EV- GPC1, MIF, and integrins served as non-invasive diagnostic and prognostic markers for the early detection of PDAC 8-10. While EV-biomarkers for discerning lymph node metastasis in PDAC are currently lacking, further research and exploration are required.

MicroRNAs (miRNAs) are small noncoding RNAs that are frequently encapsulated in EVs, imparting specific biological functions, such as promoting tumor growth, angiogenesis, and metastasis 11. In PDAC, hypoxia-induced EV-miR-301a contributed to M2 polarization of macrophages and subsequent malignant behavior 12. EV-miR-485-3p could suppress PDAC metastasis by targeting PAK1 13. However, there is still a lack of in-depth research on EV-miRNAs associated with lymph node metastasis in PDAC.

In the present study, plasma EVs were extracted from patients with N0 or N2 stage PDAC to identify potential landmarks for lymph node metastasis and to elucidate the underlying mechanisms. Subsequently, miRNA sequencing was performed on the plasma-derived EVs, and miR-205-5p was identified. The function and mechanism of miR-205-5p-mediated lymphatic metastasis were also explored.

## Materials and Methods

### Clinical samples and public databases

Fourteen peripheral blood samples were collected from patients with PDAC at the Peking Union Medical College Hospital from April 2022 to December 2022. Inclusive criteria: confirmed malignant histopathological results of the probable tumor tissues; absence of regional lymph node metastasis (N0 stage) or presence of more than 4 regional lymph node metastases (N2 stage). Exclusion criteria: Blood samples carrying hazardous pathogens or those that had undergone hemolysis before further treatment. All the patients signed an informed consent form for the use of blood samples. This study was conducted in accordance with the guidelines of the Peking Union Medical College Ethical Committee (#JS-3349).

Peripheral blood samples were centrifuged at 3000 rpm for 10 min to obtain the upper plasma, followed by EV extraction. MIR205HG expression in pancreatic cancer or normal pancreatic tissues was obtained from The Cancer Genome Atlas (TCGA)(https://portal.gdc.cancer.gov) and Genotype-Tissue Expression (GTEx)(https://www.gtexportal.org) databases.

### miRNA sequencing and bioinformatic analysis

The miRNA sequencing (miRNA-seq) of EVs was performed by Illumina HiSeq2500 platform. Bowtie software was used to align unannotated reads with the reference genome and obtain positional information on the reference genome, known as Mapped Reads. Further, by comparing the reads to the reference genome with known miRNA precursor sequences in the miRBase database, we identified the expression of known miRNAs. The TPM algorithm was used to normalize the expression level. Differential analysis was performed with edgeR software. The differentially expressed miRNAs were screened by fold change > 1.5 and *P* < 0.05.

### Tissue microarray (TMA) and *in situ* hybridization (ISH) assay

To elucidate the expression of miR-205-5p in human PDAC tissues, a tissue microarray (TMA) was utilized, which included 80 human PDAC tissues and corresponding adjacent normal pancreatic tissues (SUPERBIOTEK, China). The PDAC tissues and adjacent normal pancreatic tissues were obtained from patients diagnosed at Peking Union Medical College Hospital. Inclusion criteria were: (1) pathologically confirmed PDAC; (2) no prior chemotherapy, radiotherapy, or immunotherapy; (3) availability of complete clinical and follow-up data. Exclusion criteria included: (1) concurrent malignancies; (2) chronic inflammatory conditions (e.g., heart disease, diabetes mellitus); (3) incomplete clinical records. An in-situ hybridization assay was used to detect the expression of miR-205-5p in the TMA. One pair of staining tissues were not included in the analysis due to the invisibility. Digoxin was used to label the RNA probes. The probe for miR-205-5p was purchased from Boster Biological Technology (USA). Briefly, ISH process includes dewaxing, antigen repair, visualization of antigen-antibody hybridization, and sealing. The detailed steps can be found in a previous study 14.

### Cell culture

The human PDAC cell lines PANC1 and BxPC-3 were purchased from the American Type Culture Collection (ATCC, USA). The PANC1 cell line was cultured in DMEM medium (HyClone, Utah, USA), and BxPC-3 cells were cultured in RPMI-1640 medium (HyClone, Utah, USA) with 10% fetal bovine serum at 37 °C with 5% CO_2_. HLEC was purchased from ScienCell and cultured in Endothelial Cell Medium (ECM, ScienCell Research Laboratories, USA) at 37 °C with 5% CO_2_. Penicillin-Streptomycin Solution (100×) (Beyotime, China) was diluted and added to the medium.

### RNA extraction and qRT‒PCR

TRIzol reagent (Invitrogen, Cat. No. 15596-026) was used to extract total cellular RNA, according to the manufacturer's protocol. RNA concentration was determined using a NanoDrop spectrophotometer (NanoDrop Technologies, ND-1000). Reverse transcription was performed using a Reverse Transcription Kit (Thermo Fisher, Cat. No. #K1622). Quantitative RT-PCR (qRT-PCR) was conducted using the PowerUp SYBR Green Master Mix (Thermo Fisher, Cat. No. A25742). Relative mRNA expression was calculated using the 2^-ΔΔCt method.

### EV collection, isolation and treatment

Size Exclusion Chromatography (SEC) was used to extract the plasma-derived EVs. Briefly, 1 mL of plasma was added to the top of the column extractor (Exosupur, Echo Biotech, China) following the manufacturer's protocol and the effluent was collected as purified EVs 15. To collect cell-derived EVs, cell supernatants were collected after culturing in FBS-free medium for 24 h. A total of 200 mL of supernatant was collected from each group. The cell supernatants were then centrifuged at 2,000 × g for 15 min to remove cell debris. Supernatants were collected and centrifuged at 100,000 × g for 70 min. The precipitates were resuspended in 5 mL of PBS and further centrifuged at 100,000 × g for 70 min. The final precipitate was resuspended in 200 μL of PBS. A noncompetitive neutral sphingomyelinase inhibitor, GW4869 (HY-19363, MCE, USA) was used to inhibit the generation and release of EVs in cells 16. The protein concentration of the EVs was determined using a BCA kit. For the co-culture system, 3 × 10^5^ tumor cells (in 6-well plates) were added to 5 μg EVs. For the subcutaneous xenograft model, 2 × 10^6^ treated cells were mixed with 30 μg of EVs (in PBS) before injection, and an additional 30 μg of EVs (in PBS) was injected into the xenografts after 2 weeks. For the popliteal lymph node metastasis model, 3 × 10^5^ tumor cells were mixed with 5 μg EVs (in PBS) before injection.

### Cell migration assay and cell co-culturing assay

Transwell chambers (8 μm, Corning, NY, USA) were used to test cell migration ability. Briefly, 4 × 10^4^ pancreatic cancer cells (or 5 × 10^4^ HLECs) in 100 μL of serum-free medium were plated on the upper part of the chamber. Next, 600 μL of the corresponding medium with 10% FBS was added to the lower chamber of each well of a 24-well plate. After culturing for 24 h at 37 °C with 5% CO_2_, the migrated cells were fixed with methanol and stained with hematoxylin and eosin (HE). The cells were imaged and recorded under a microscope.

Transwell chambers (0.4 μm, Corning, NY, USA) were used to perform cell co-culturing. The HLECs were plated on the lower chamber of each well of a 24-well plate. And the donor cells (PANC1) were plated on the upper part of the chamber. After co-culturing for 48 h, the RNA and proteins of HLECs were extracted for further verification.

### Cell proliferation assay

Cell proliferation was detected using the Sulforhodamine B (SRB) dyeing method. Briefly, 4000 treated cells were plated in each well of a 96-well plate. After cell attachment or culturing for 24, 48, 72, and 96 h, the cells were fixed with a 10% trichloroacetic acid (TCA) water solution and stained with a 1% SRB acetic acid water solution. The cells were then washed twice with 1% acetic acid-water solution. Finally, 10% Tris-base water solution was used to dissolve the SRB. Optical density (OD) at 564 nm was measured using a spectrophotometer. The cell proliferation rate was determined by comparing the OD values at the corresponding time points.

### Cell transfection

The MIR205 mimics (RiboBio, China) were used to enhance the regulatory role of miR-205-5p. The MIR205 inhibitor (RiboBio, China) was used to inhibit the function of miR-205-5p. The VEGFA-overexpression plasmid was used to overexpress VEGFA mRNA. Lipo8000 (C0533, Beyotime, China) and Opti-MEM™ (31985062, Invitrogen, USA) were used to transfect miRNA mimics, inhibitors, or plasmids, according to the manufacturer's instructions. MIR205-up lentivirus (Genechem, China) was used to stably express pre-miR-205-5p. HiTransG P Reagent (Genechem, China) was used to enhance transfection efficiency.

### Western blot and antibodies

Cellular protein was extracted using RIPA lysis buffer (P0013B, Beyotime, China) following the manufacturer's instructions, and the protein concentration was measured using a BCA kit (P0012, Beyotime, China). Western blotting experiments were conducted according to a previous study 17. The primary antibodies used were as follows: anti-VEGFA antibody (19003-1-AP, Proteintech, China); anti-vinculin antibody (66305-1-Ig, Proteintech, China); Anti-ERK1/2 antibody (11257-1-AP, Proteintech, China); Anti-Phospho-ERK1/2 (28733-1-AP, Proteintech, China); anti-AKT antibody (60203-2-Ig, Proteintech, China); anti-phospho-AKT (Ser473) antibody (80455-1-RR, Proteintech, China); anti-calnexin antibody (ab22595, Abcam, China); anti-Alix antibody (ab275377, Abcam, China); Anti-TSG101 antibody (ab125011, Abcam, China); and Anti-CD63 antibody (ab134045, Abcam, China).

### PKH-67 staining assay

PKH-67 (MINI67-1KT, Sigma, USA) was used to stain the membranes of EVs and illustrate the uptake of EVs by cells. The purified EVs in 150 μL of PBS were mixed with PKH-67 (diluted in 150 μL of Diluent C) for 5 min and then terminated by mixing with 3% BSA. The labeled EVs were washed with PBS and further purified by ultracentrifugation as described above. The labeled EVs were resuspended in PBS and co-cultured with HLECs for 24 h. DAPI (C1002, Beyotime, China) staining was performed the following day. Fluorescence was detected and photographed using a confocal microscope.

### Enzyme-linked immunosorbent assay (ELISA)

The expression levels of VEGFA in the cell supernatant were quantified using an ELISA kit (Abcam, ab119566), following the manufacturer's protocol. Briefly, the cell supernatant was added to the wells of plates embedded with the primary capture antibody. After each incubation, the wells were washed three times. Anti-human VEGFA antibody and Streptavidin-HRP Conjugate were successively added to each well. Finally, TMB Substrate was added to each well. The plates were then incubated at room temperature. Optical density (OD) was recorded using a microplate reader at 450 nm. The VEGFA protein concentration was calculated using a standard curve.

### Tube formation assay

Lymphatic vessel formation by HLEC cells was assessed using the tube formation method. Next, 200 μL of BD Matrigel (356234, Corning, USA) was added to the bottom of each 24-well plate and incubated with 5% CO_2_ at 37 °C for 30 min. Next, 4 × 10^4^ treated cells in 200 μL of cell medium were seeded on the surface of the coagulated matrix gel. Tube structures emerged after six hours of cultivation. The images of the tubes were examined and captured under a microscope.

### *In vivo* experiments

BALB/c nude mice (4 weeks, female) were purchased from Vitalstar Biotechnology Co., Ltd. (Beijing, China). To elucidate the role of miR-205-5p *in vivo*, a subcutaneous xenograft model and a popliteal lymph node metastasis model were established in nude mice. The mice were anesthetized by intraperitoneal injection of 1.25% tribromoethanol (20 μL/g) before injection. The former model was established by subcutaneous injection of 3 × 10^6^ treated cells in 100 μL of PBS. A popliteal lymph node metastasis model was established by injecting 3 × 10^5^ treated cells into the footpads. Each group contained 6 nude mice. The mice were monitored every 2 days and if the average tumor diameter exceeded 20 mm, a humane endpoint would be applicable. No mice have met the above standards or died before 3 weeks. The mice were sacrificed in a CO_2_ chamber after 3 weeks. We try to minimize the pain of mice as much as possible during the operation and took further action after confirming the death of the animal. The xenografts were separated and weighed. The volume of the lymph nodes was separated and calculated by multiplying the length by the square of the width. The xenograft and lymph node samples were further embedded and cut into slices for IHC or HE/ISH staining. All animal experiments were conducted in accordance with the guidelines of the Institutional Animal Care and Use Committee (Beijing, China).

### Statistical analysis

All data are presented as mean values with standard errors. Student's t-test or one-way ANOVA was used to compare the statistical differences between groups. *P* < 0.05 was considered statistically significant. All functional experiments were conducted at least three times. Diagrams and images were generated using the Prism 8 software.

## Results

### Screening of key EV-miRNA for lymph node metastasis in PDAC

**Figure 1:**
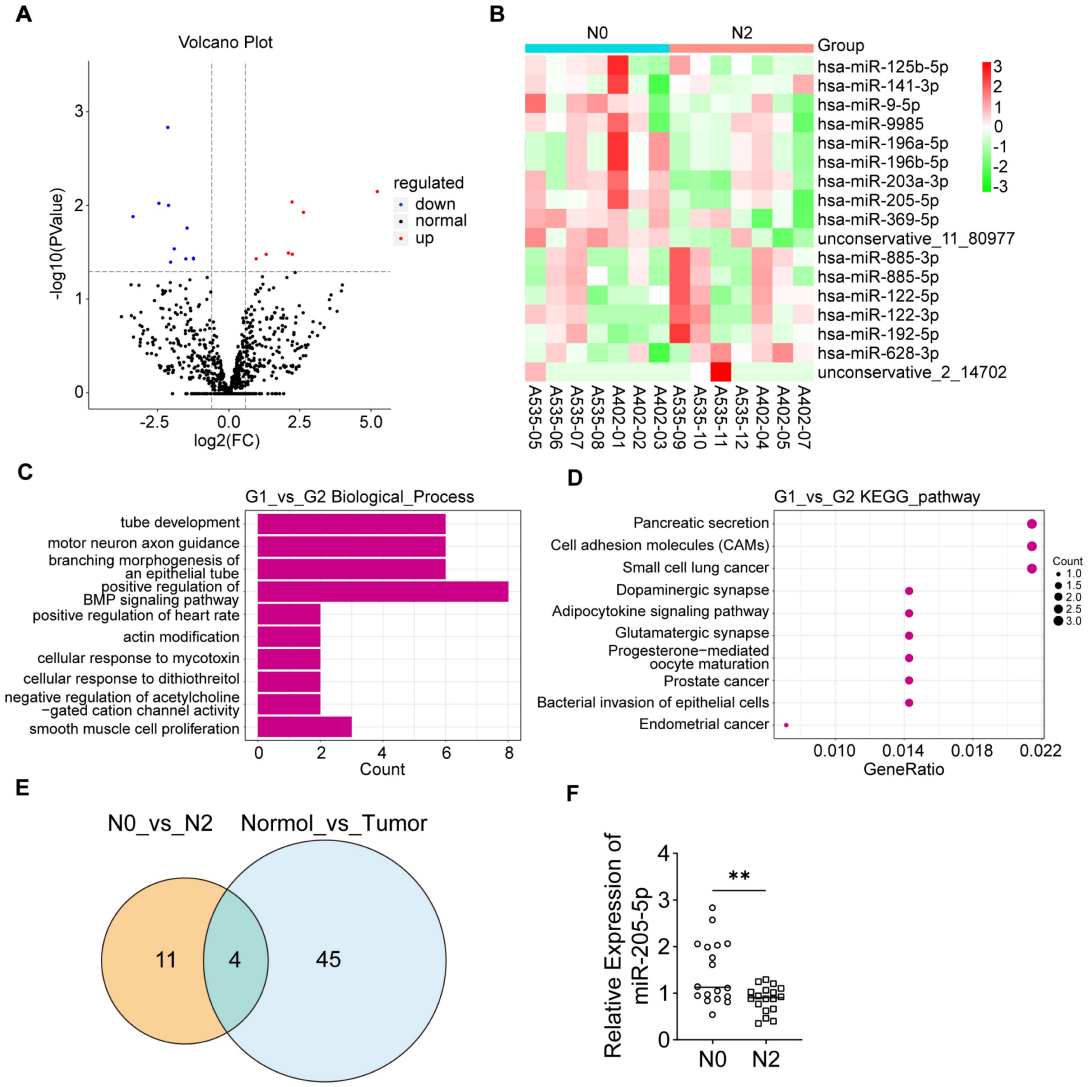
** Screening of key EV-miRNAs for lymph node metastasis in pancreatic cancer.** (A) A volcano plot that showed differentially expressed miRNAs with statistical significance and log2 fold change in the N2 group compared to N0 group. (B) A clustering heatmap demonstrated the relative change and names of miRNAs between the N2 group compared to N0 group. (C) GO functional analysis of predicted target genes showed enrichment in the biological process of tube development, branching morphogenesis of an epithelial tube that relate to the lymphangiogenesis and so on. (D) KEGG pathway analysis of the predicted target genes showed correlation with Pancreatic Secretion, Cell adhesion molecules (CAMs), etc. (E) Venn chart showed the intersection of differentially expressed EV-miRNAs in PDAC (N0 stage versus N2 stage) and differentially expressed miRNAs in PDAC tumor tissue compared to peri-tumor tissue. (F) The expression of EV-miR-205-5p was verified in other patients with PDAC (6 of N0 stage and 6 of N2 stage). **: *P* < 0.01.

To elucidate the role of EV-miRNAs in PDAC lymph node metastasis, peripheral blood samples were obtained from 14 patients with PDAC (7 at N0 stage and 7 at N2 stage; Supplementary Data 1). Subsequently, the plasma EVs were isolated for analysis. The positive EV-protein markers Alix, TSG101, and CD63 were highly expressed in both N0 and N2 stage plasma-derived EVs, and the negative EV-protein marker calnexin was barely expressed in EVs but was highly expressed in the cell lysate (Supplementary Figure 1A). Nanoparticle size analysis (NTA) of the plasma EVs revealed typical EV diameters (Supplementary Figure 1B). miRNA sequencing of plasma EVs from patients with different lymph node status was performed. The volcano plot displayed several differentially expressed miRNAs (Figure 1A). A heatmap plot showed that miR-125b-5p, miR-141-3p, miR-9-5p, miR-9985, miR-196a-5p, miR-196b-5p, miR-203a-3p, miR-205-5p, and miR-369-5p were upregulated in the N0 group, whereas miR-885-3p, miR-885-5p, miR-122-5p, miR-122-3p, miR-192-5p, and miR-628-3p were upregulated in the N2 group (Figure 1B). GO functional enrichment analysis of predicted target genes for differentially expressed miRNAs revealed that the genes were mostly enriched in the biological processes of tube development and branching morphogenesis of an epithelial tube, which are related to lymphangiogenesis (Figure 1C). KEGG analysis of predicted target genes for differentially expressed miRNAs indicated the relevance of Pancreatic Secretion, Cell adhesion molecules (CAMS), and so on (Figure 1D). GO analysis showed enrichment of the biological processes of cell aggregation and cellular components of the extracellular matrix in differentially expressed miRNA targets compared to all miRNA targets (Supplementary Figure 1C). We wondered whether these EV-miRNAs were actually present in tumor tissues. Therefore, the differentially expressed EV-miRNAs in PDAC (N0 stage versus N2 stage) were intersected with the differentially expressed miRNAs in PDAC tumor tissue compared to peri-tumor tissue in another study 18. Four miRNAs were screened, among which miR-205-5p showed the most significant difference (Figure 1E). These results indicated that miR-205-5p was highly expressed in both PDAC tissue and N0 stage PDAC plasma-derived EVs. Further verification in other patients with PDAC (six at N0 stage and six at N2 stage) showed that plasma EV-miR-205-5p was highly expressed in N0 stage PDAC compared to N2 stage PDAC (Figure 1F).

### miR-205-5p was highly expressed in PDAC but negatively associated with lymph node metastasis

**Figure 2:**
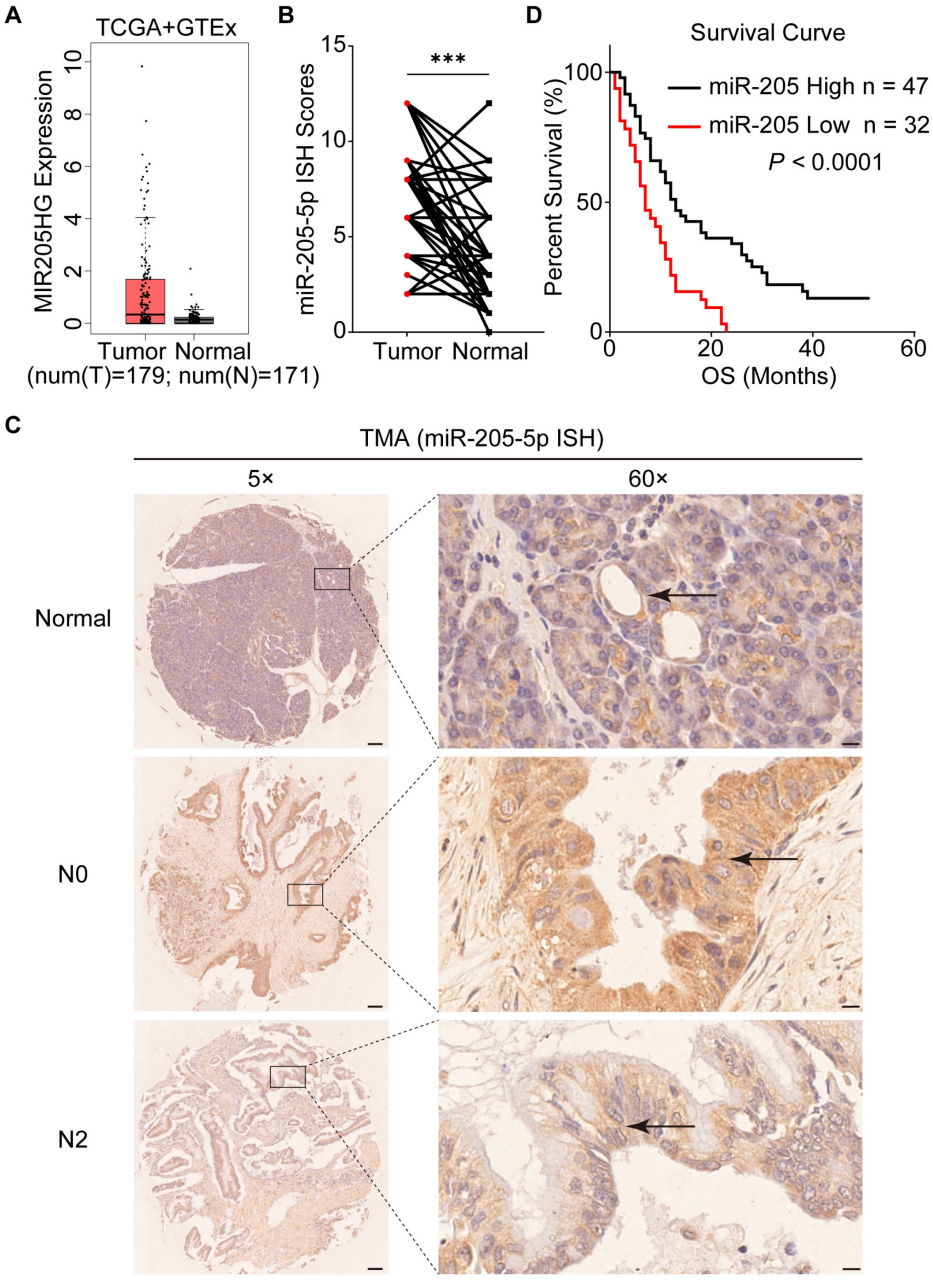
** MiR-205-5p expression was negatively correlated with lymph node metastasis.** (A) The expression of MIR205HG in pancreatic cancer tissues and normal pancreatic tissues from the TCGA and GTEx database. (B) The ISH score of miR-205-5p in TMA (n = 79). (C) Representative images of ISH results for miR-205-5p in adjacent normal pancreatic tissue, N0 stage PDAC and N2 stage PDAC. The arrows indicated the normal pancreatic duct or the pancreatic cancer. Scale bars: 100 μm (left). Scale bars: 10 μm (right). (D) The Kaplan-Meier survival curve of patients with high or low expression of miR-205-5p from the TCGA database (*P* < 0.0001). ***: *P* < 0.001.

To explore the role of EV-miR-205-5p in PDAC, the expression of MIR205HG (miR-205-5p precursor) in PDAC tissue was obtained from the TCGA database. The box plot showed that MIR205HG was expressed at higher levels in tumor tissues than in normal adjacent tissues (Figure 2A). A total of 79 PDAC patients were included in the TMA analysis to assess the expression of miR-205-5p in cancer and normal tissues. ISH results of the TMA showed that the expression of miR-205-5p in PDAC tissues was significantly higher than that in normal pancreatic tissues (Figure 2B). Representative ISH images of miR-205-5p in tumor and normal adjacent tissues are shown in Figure 2C. By comparing the expression of miR-205-5p, patients were divided into high and low expression groups. Patients with high levels of miR-205-5p displayed more N0 (*P* = 0.000) and I/IIA stages (*P* = 0.000) (Table 1). Univariate and multivariate analyses revealed that low differential degree (*P* = 0.000), M1 stage (*P* = 0.036), and low miR-205 expression (*P* = 0.014) were independent prognostic risk factors for patients with PDAC (Table 2). The survival curve indicated that patients with high miR-205-5p expression had prolonged survival times, suggesting that miR-205-5p may have a tumor inhibitory effect (Figure 2D). These results indicated elevated expression of miR-205-5p in PDAC tissues and its potential anti-tumor effect in the development and progression of this disease.

### MiR-205-5p inhibited migration and proliferation in PDAC cell lines

**Figure 3:**
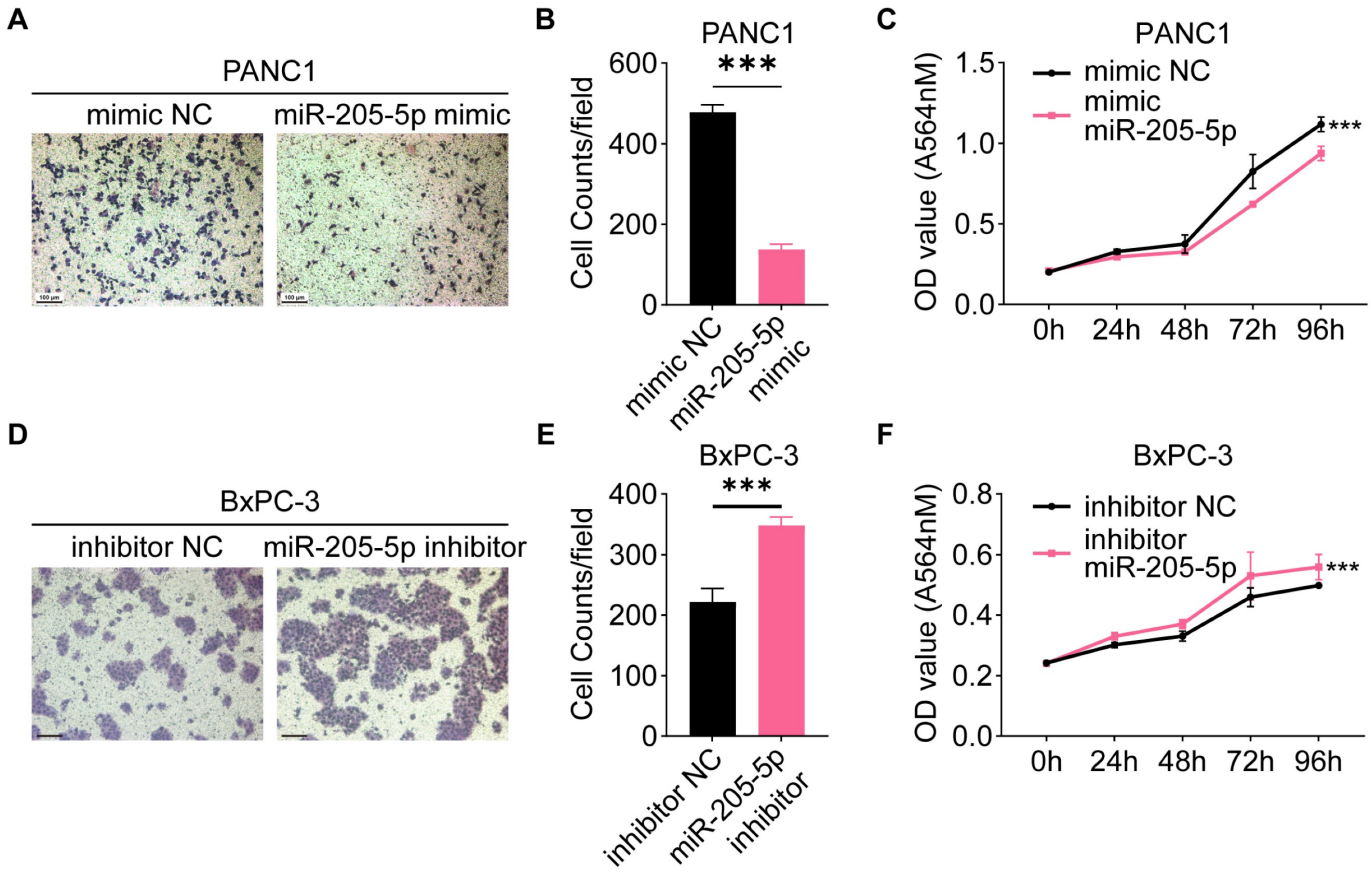
** MiR-205-5p inhibited migration and proliferation in pancreatic cancer cell lines.** (A) The cell migration ability was tested after mimic miR-205-5p in PANC1. (B) Statistical chart of the cell migration ability in PANC1. (C) The cell proliferation ability was tested after mimic miR-205-5p in PANC1. (D) The cell migration ability was tested after inhibition of miR-205-5p in BxPC-3. (E) Statistical chart of the cell migration ability in BxPC-3. (F) The cell proliferation ability was tested after inhibition of miR-205-5p in BxPC-3. Scale bars: 100 μm. ***: *P* < 0.001.

Since we tentatively identified a negative correlation between miR-205-5p and lymph node metastasis in TMA, we investigated the specific role of miR-205-5p in PDAC. First, the expression of miR-205-5p was tested in a normal pancreatic ductal cell line (HPNE), several PDAC cell lines (AsPC-1, BxPC-3, CFPAC, MIA PaCa-2, and PANC1), and HLEC. The results indicated that the expression of miR-205-5p was relatively low in HPNE, PANC1, and HLEC, but relatively high in BxPC-3 cells (Supplementary Figure 1D). Overexpression of miR-205-5p in PANC1 (Supplementary Figure 1E) and inhibition of miR-205-5p in BxPC-3 cells, migration, and proliferation abilities were tested. The results showed impaired migration and proliferation ability after overexpression of miR-205-5p in PANC1 cells (Figure 3A, B, and C) and enhanced migration and proliferation ability after inhibition of miR-205-5p in BxPC-3 cells (Figure 3D, E, and F). These *in vitro* results revealed that miR-205-5p inhibited the migration and proliferation of PDAC cells.

### EV-miR-205-5p was transferred to the HLEC cell to attenuate migration and lymphangiogenesis

**Figure 4:**
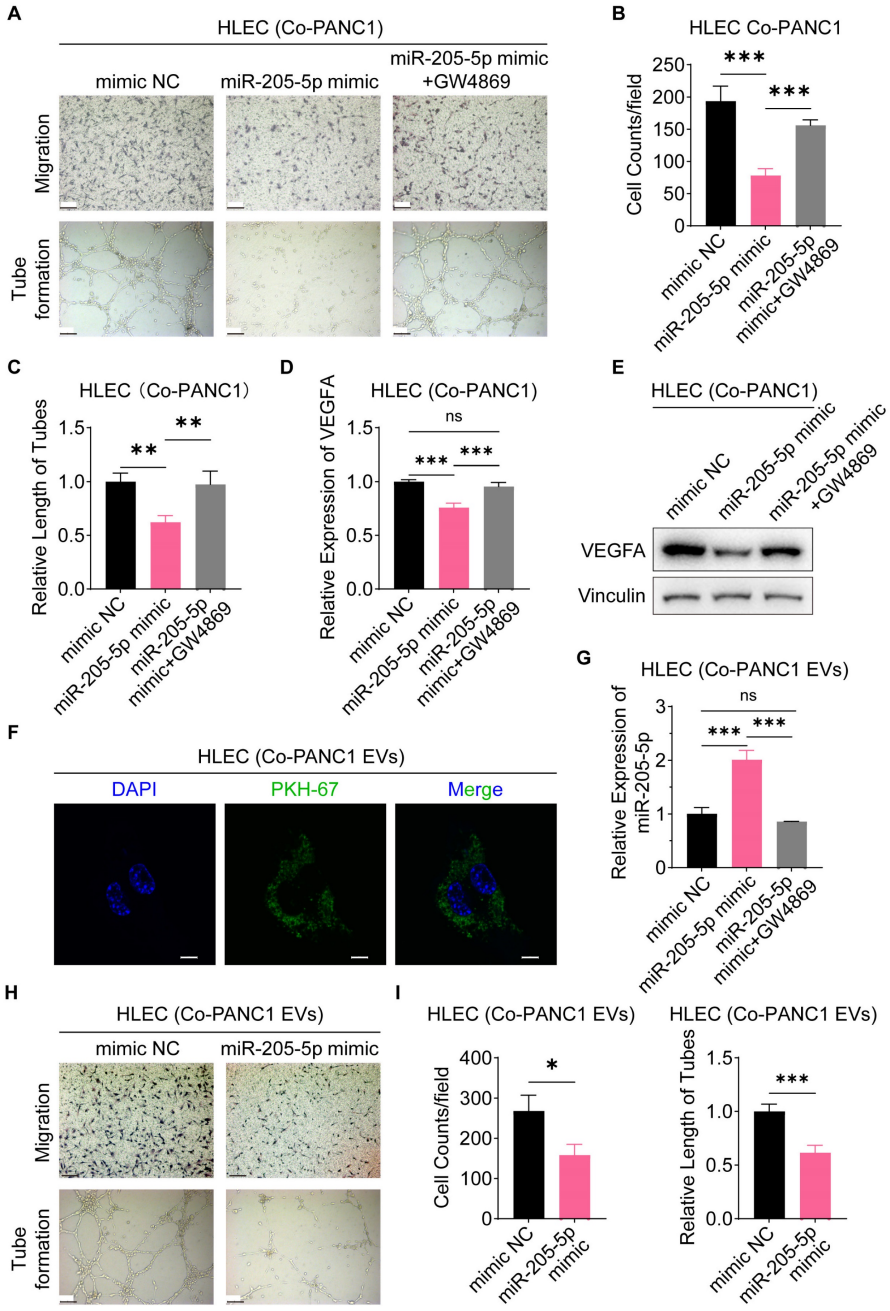
**EV-miR-205-5p was transferred to HLEC cell line to attenuate migration and lymphangiogenesis.** (A-C) The cell migration ability and tube formation ability of HLEC were tested after co-cultured with miR-205-5p-overexpressed PANC1-EVs and further adding GW4869. Scale bar: 100 μm. (D-E) The RNA and protein expression of VEGFA in HLEC was tested after co-cultured with miR-205-5p-overexpressed PANC1-EVs and further adding GW4869. (F) Representative immunofluorescence images showed internalization of PKH-67-stained EVs (green) in HLEC. Scale bars: 10 μm. (G) The mRNA expression of miR-205-5p in HLEC was tested after co-cultured with miR-205-5p-overexpressed PANC1 and further adding GW4869. (H-I) The cell migration ability and tube formation ability were tested in HLEC after co-cultured with miR-205-5p-overexpressed PANC1-EVs. Scale bar: 100 μm. *: *P* < 0.05. **: *P* < 0.01. ***: *P* < 0.001.

Furthermore, we investigated the role of EV-miR-205-5p in lymph node metastasis *in vitro*. Lymphangiogenesis is the rate-limiting step and enables lymph node metastasis in PDAC. Therefore, migration and tube formation of HLECs were examined after co-culturing with PDAC cells. When co-cultured with miR-205-5p overexpressed-PANC1 cells, the number of migrated HLECs decreased and the tube formation ability of HLECs was also impaired, while the number of migrated HLECs returned to baseline levels when an EV release inhibitor, GW4869, was added, along with the restoration of tube formation ability (Figure 4A, B, and C). At the same time, the RNA and protein expression of VEGFA was downregulated in HLECs after co-cultured with miR-205-5p overexpressed-PANC1 cells and restored after the addition of GW4869 (Figure 4D, E). To verify whether the functional changes in HLECs were affected by PANC1-secreted EVs, PANC1-secreted EVs were labeled with green fluorescence using PKH-67 and co-cultured with HLECs. Green fluorescent-labeled EVs were clearly ingested by the HLECs (Figure 4F). The expression of miR-205-5p in HLEC was upregulated after co-culturing with miR-205-5p overexpressed-PANC1 EVs and downregulated after adding GW4869 (Figure 4G). Finally, PANC1-secreted EVs and the miR-205-5p overexpressed-PANC1-secreted EVs were extracted and co-cultured separately with HLECs. The number of migrated HLECs was reduced, and the tube formation ability of HLECs was impaired (Figure 4H, I). The miR-205-5p overexpressed-PANC1-secreted EVs also inhibited the growth of HLECs (Supplementary Figure 1F). These results illustrate that EV-miR-205-5p from cancer cells could attenuate the migration and tube formation ability of HLECs.

### VEGFA was the direct target of miR-205-5p

**Figure 5:**
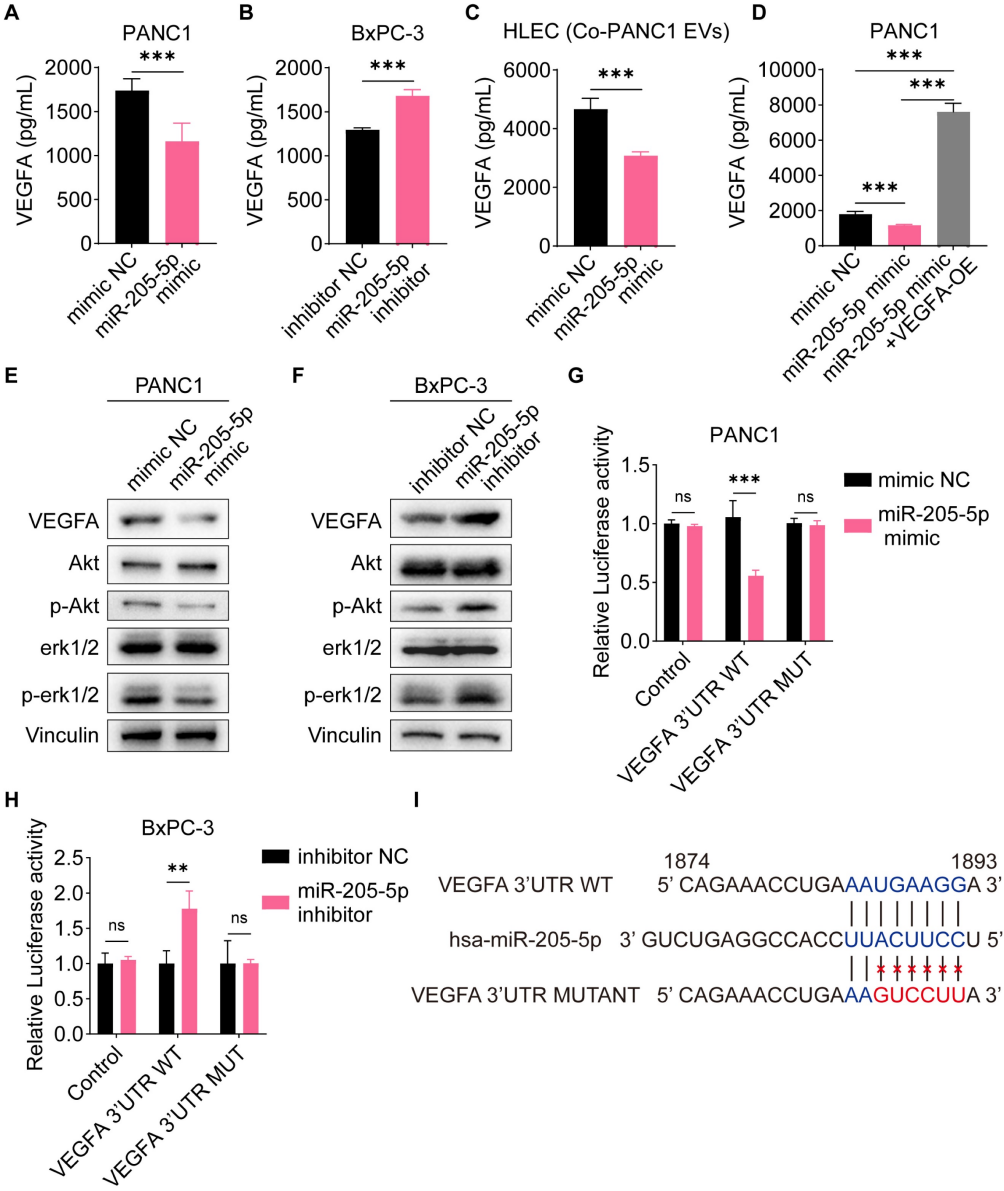
** VEGFA was the direct target of miR-205-5p.** (A-B) The concentration of cell secretory VEGFA was compared after mimic miR-205-5p in PANC1 and inhibition of miR-205-5p in BxPC-3. (C) The concentration of cell secretory VEGFA in HLEC was compared after co-cultured with miR-205-5p-overexpressed PANC1-EVs. (D) The concentration of cell secretory VEGFA was compared after mimic miR-205-5p in PANC1 and further overexpression of VEGFA. (E-F) The expression of VEGFA and downstream molecules of VEGFA were tested after mimic miR-205-5p in PANC1 (E) or inhibition of miR-205-5p in BxPC-3 (F). (G-H) The relative luciferase activity was tested after overexpression of VEGFA 3' UTR WT plasmid or VEGFA 3' UTR MUT plasmid in PANC1 (G) and BxPC-3 (H). **: *P* < 0.01. ***: *P* < 0.001. (I) A summary diagram showing the putative target sites of miR-205-5p-VEGFA and the mutant sites of dual-luciferase reporter assay.

To identify the direct targets of miR-205-5p, we screened several potential targets of miR-205-5p from previously published studies, including VEGFA, FGF1, ANGPT2, CDK6, TNFAIP8, and ERBB3. The mRNA expression of these potential targets was tested after overexpression of miR-205-5p in PANC1 cells and inhibition of miR-205-5p in BxPC-3 cells (Supplementary Figure 2A, B). Among the multiple candidates, only VEGFA was downregulated after overexpression of miR-205-5p in PANC1 and upregulated after inhibition of miR-205-5p in BxPC-3 cells (Supplementary Figure 2A, B). As VEGFA is a secretory protein, the concentration of VEGFA in the cell supernatant was tested. The concentration of VEGFA in the cell supernatant was significantly reduced following the overexpression of miR-205-5p in PANC1 cells and increased after the inhibition of miR-205-5p in BxPC-3 cells (Figure 5A, B). Furthermore, the concentration of VEGFA in the HLEC supernatant decreased after co-cultured with miR-205-5p-overexpressed PANC1-secreted EVs (Figure 5C). Secreted VEGFA protein was upregulated after transfection with the VEGFA-overexpression plasmid in PANC1 cells (Supplementary Figure 2C). The results showed that the downregulation of secreted VEGFA protein after the overexpression of miR-205-5p in PANC1 could be counteracted by VEGFA overexpression (Figure 5D). As both the Akt and Erk pathways are downstream effectors of VEGFA-VEGFR2 signaling, we therefore assessed the activation of these pathways 19. The phosphorylation of Akt and erk1/2 was diminished after overexpression of miR-205-5p in PANC1 cells and upregulated after inhibition of miR-205-5p in BxPC-3 cells (Figure 5E, F). Finally, the putative target site of miR-205-5p targeting VEGFA was predicted by starBase v2.0 (https://rnasysu.com/encori/index.php) and dual-luciferase reporter gene assay was performed. The relative luciferase activity was downregulated after transfection of the VEGFA wild-type plasmid and overexpression of miR-205-5p in PANC1 cells; however, it was upregulated after the inhibition of miR-205-5p in BxPC-3 cells (Figure 5G, H). These results confirm the regulation of VEGFA by miR-205-5p and the direct binding of miR-205-5p to VEGFA mRNA (Figure 5I).

### EV-miR-205-5p affected cell migration and lymphangiogenesis via the VEGFA pathway

**Figure 6:**
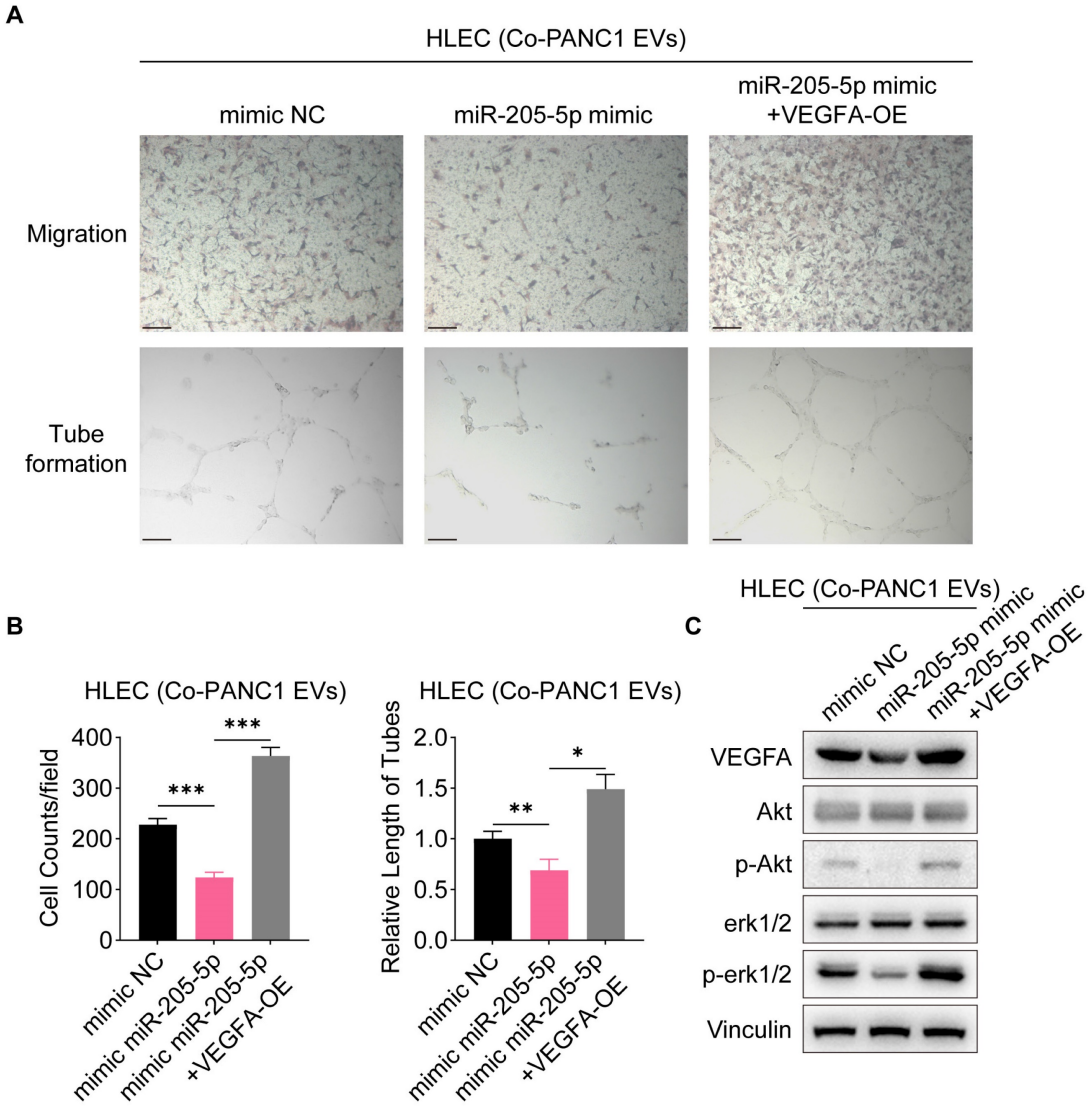
** MiR-205-5p affected cell migration and lymphangiogenesis via VEGFA pathway.** (A-B) The cell migration ability and tube formation ability of HLEC were tested after co-cultured with miR-205-5p-overexpressed PANC1-EVs and further overexpression of VEGFA. (C) The expression of VEGFA and its downstream molecules were tested after co-cultured with miR-205-5p-overexpressed PANC1-EVs and further overexpression of VEGFA in HLEC. Scale bars: 100 μm. *: *P* < 0.05. **: *P* < 0.01. ***: *P* < 0.001.

We further tested and verified the effectiveness of EV-miR-205-5p by VEGFA overexpression. The number of migrated HLECs decreased and the tube formation ability of HLECs was also impaired after co-culture with the miR-205-5p-overexpressed EVs, however, the number of migrated HLECs was restored with the transfection of the VEGFA-overexpression plasmid, along with the restoration of tube formation ability (Figure 6A, B). The phosphorylation of Akt and erk1/2 was clearly impeded after the addition of miR-205-5p-overexpressed PANC1-secreted EVs but was restored after overexpression of VEGFA (Figure 6C; Supplementary Figure 2D). These results indicated that EV-miR-205-5p exerted its effects by blocking the VEGFA pathway.

### EV-miR-205-5p affected cell proliferation and lymphangiogenesis *in vivo*

**Figure 7:**
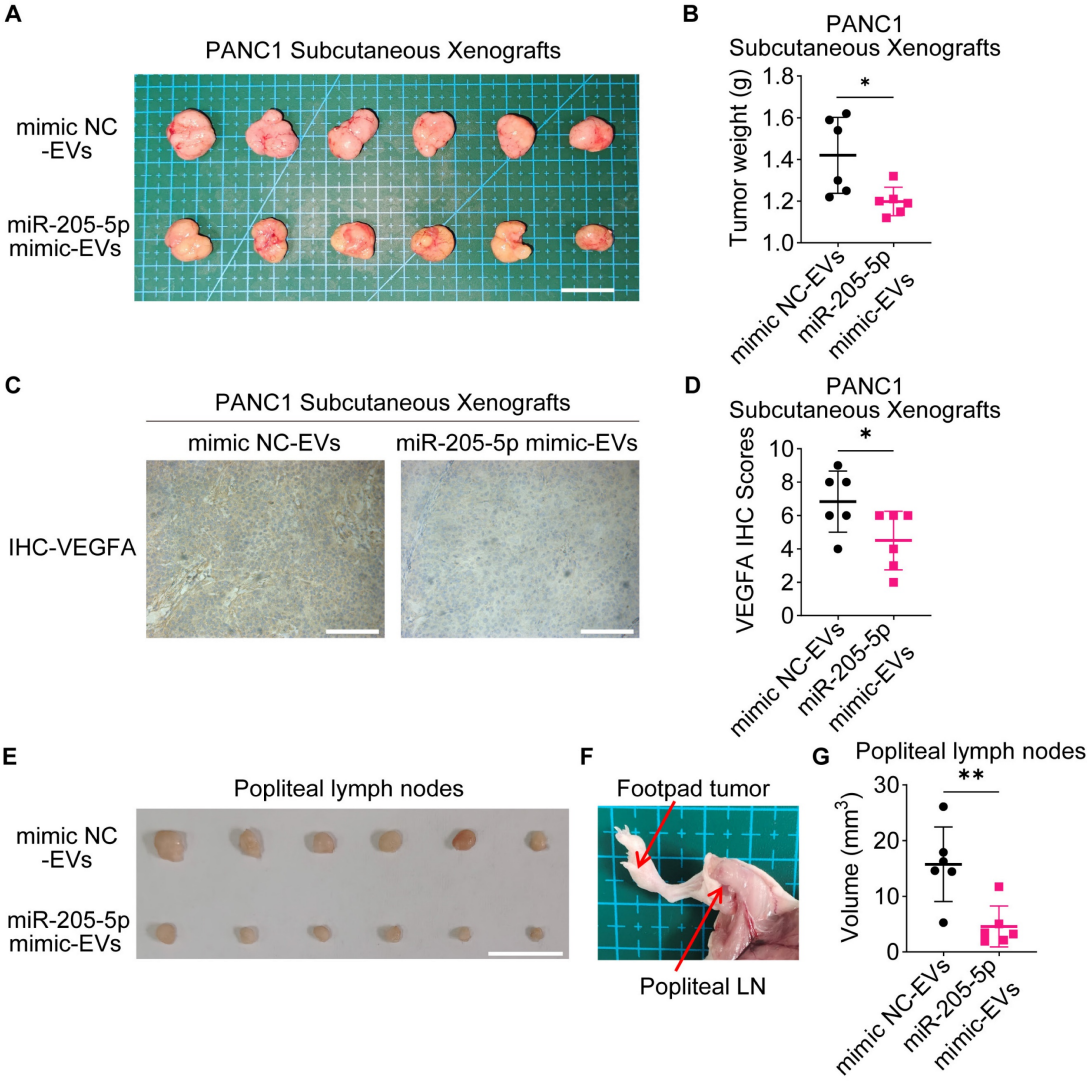
**EV-miR-205-5p affected migration and lymphangiogenesis *in vivo*.** (A-B) Subcutaneous xenografts of PANC1 with CON-EVs or miR-205-5p-overexpressed EVs (A) were shown and the tumor weight were measured (B). Scale bar: 2 cm. (C) Representative images of VEGFA IHC staining results in subcutaneous xenografts of PANC1 with CON-EVs or miR-205-5p-overexpressed EVs were shown. Scale bars: 100 μm. (D) The VEGFA IHC scores in subcutaneous xenografts of PANC1 with CON-EVs or miR-205-5p-overexpressed EVs were shown. (E) Popliteal lymph nodes between the groups of PANC1 with CON-EVs or miR-205-5p-overexpressed EVs were compared. Scale bar: 1 cm. (F) A diagram showed the footpad tumor and the popliteal lymph node. (G) The volumes of popliteal lymph nodes in were compared.

To further investigate the role of EV-miR-205-5p in tumor growth *in vivo*, PANC1 cells with EVs were subcutaneously transplanted into nude mice. The addition of miR-205-5p-enriched EVs slowed the tumor growth rate (Figure 7A, B) and reduced the expression of VEGFA (Figure 7C, D). PANC1 cells with different sources of EVs were injected into the footpads of mice to observe the lymph node metastasis status. The swelling of the popliteal lymph nodes in the experimental group was significantly smaller than that in the control group (Figure 7E, F, and G). The HE staining results showed more deeply stained lymphocytes and more normal structures in the swollen lymph nodes of the experimental group (Supplementary Figure 2E). These results indicate that EV-miR-205-5p weaken PDAC growth and simultaneously impair lymph node metastasis.

## Discussion

MiR-205-5p has been found to have diverse effects in multiple types of cancer. In breast cancer, miR-205-5p has shown promising results by inhibiting cell proliferation and impairing the ability to invade the surrounding tissues 20-22. Similarly, in prostate cancer, miR-205 blocked orthotopic tumor growth 23. However, in ovarian cancer, it exhibited a pro-metastasis effect by inducing tumor angiogenesis and served as a potential therapeutic target 24, 25. In the current study, we uniquely correlated EV-miR-205-5p levels with N0 stage in PDAC patients, positioning it as a potential prognostic biomarker. EV-miR-205-5p may be transferred from cancer cells to HLECs, thereby blocking the secretion of VEGFA and inhibiting lymphatic metastasis. We emphasized the dual downregulation of VEGFA in both tumor cells and HLECs via EV-miR-205-5p transfer, a mechanism not previously described. Thus, EV-miR-205-5p could be promising in the treatment of PDAC.

VEGFA is secreted by various cells, such as endothelial cells and tumor cells, and was initially proven to be an endothelial growth factor and a regulatory factor for vascular permeability 19, 26. VEGFA can promote the growth of endothelial cells in the arteries, veins, and lymphatic vessels. By binding to the VEGF receptor (VEGFR), VEGFA activates multiple intracellular signaling pathways, including the PI3K/Akt and MAPK/ERK pathways 27, 28. Typically, VEGFC with the ligand VEGFR3 was thought to be the key regulator of lymphangiogenesis 29. However, we did not find the connection of miR-205-5p and VEGFC. A previous revealed that VEGFA could induces active proliferation of VEGF receptor-2-expressing tumor-associated lymphatic vessels as well as tumor metastasis to the sentinel and distant lymph nodes 30. Given that VEGFA plays a crucial role in regulating angiogenesis and lymphangiogenesis during the development of solid tumors, targeted therapy for VEGFA has emerged as a novel treatment approach in oncology. In the current study, VEGFA was targeted by miR-205-5p in both tumor and endothelial cells. More importantly, miR-205-5p could be encapsulated in EVs and stably transferred from cancer cells to HLECs.

VEGFA has been shown to be associated with immune response, but this was not the focus of our study. VEGFA signaling was found to be directly involved in Treg cell regulation and correlated with the expression of inhibitory receptors, including PD-1 on CD8^+^ CTL 31. In addition, transcriptomic data revealed a negative correlation between the level of VEGFA and the infiltration characteristics of CD8^+^ CTL in biopsy tissues of patients with breast cancer and in preclinical models of breast cancer and colorectal cancer 32, 33. In an ovarian cancer mouse model, resistance to VEGFA-targeted antibodies was associated with the accumulation of immunosuppressive cells, including tumor-associated macrophages (TAM) 34. VEGFA similarly exhibits immune resistance in PDAC, and this immunosuppressive effect, inhibited by EV-miR-205-5p, should be further explored in the future 35.

Numerous miRNAs have been documented to play crucial roles in the biological processes of cancer, including proliferation, migration, angiogenesis, and lymphangiogenesis. In this study, we found that plasma EV-miR-205-5p was highly expressed in N0-stage PDAC patients. At the tissue level, miR-205-5p was highly expressed in PDAC tissues, specifically in the N0 stage, rather than in adjacent normal pancreatic tissue or N2-stage PDAC. We further verified the uptake of EVs by HLEC and the role of EV-miR-205-5p both *in vitro* and *in vivo*. Finally, VEGFA was proved the mediator that inhibited PDAC progression. Overall, this study provides compelling evidence that VEGFA is a direct target of miR-205-5p, shedding light on the intricate regulatory pathways involved in angiogenesis and offering new possibilities for therapeutic intervention.

## Supplementary Material

Supplementary figures and data legend.

Supplementary data.

## Figures and Tables

**Table 1:** The expression of miR-205 and clinical characteristics of patients.

		miR-205 expression		
Variables	n	Low group n=32	High group n=47	P value
Gender				0.716
Male	40	17	23	
Female	39	15	24	
Age				0.986
<60	32	13	19	
≥60	47	19	28	
Location				0.653
Head	50	20	30	
Body/tail	29	12	17	
Differential degree				0.101
Low	43	21	22	
High/moderate	36	11	25	
T stage				0.359
T1/T2	54	20	34	
T3/T4	25	12	13	
N stage				0.000
N0	49	12	37	
N1/N2	30	20	10	
M stage				0.175
M0	67	25	42	
M1	12	7	5	
TNM stage				0.000
I/IIA	40	8	32	
IIB/III/IV	39	24	15	

**Table 2:** Univariate and multivariate analyses of prognostic risk factors.

		Univariate		Multivariate	
Variables	n	HR (95% CI)	P value	HR (95% CI)	P value
Gender			0.341		
Male	40	1			
Female	39	0.796 (0.497~1.274)			
Age			0.910		
<60	32	1			
≥60	47	0.973 (0.608~1.557)			
Location			0.334		
Head	52	1			
Body/tail	27	0.783 (0.477~1.286)			
Differential degree			0.000		0.000
High/moderate	43	1		1	
Low	36	3.201 (1.962~5.223)		3.207 (1.899~5.418)	
T stage			0.179		
T1/T2	54	1			
T3/T4	25	1.405 (0.856~2.307)			
N stage			0.000		0.105
N0	49	1		1	
N1/N2	30	2.562 (1.542~4.259)		2.360 (0.835~6.667)	
M stage			0.003		0.036
M0	67	1		1	
M1	12	2.643 (1.394~5.012)		2.729 (1.066~6.991)	
TNM stage			0.000		0.840
I/IIA	40	1		1	
IIB/III/IV	39	2.542 (1.554~4.158)		0.893 (0.297~2.680)	
miR-205 expression			0.000		0.014
Low	32	1		1	
High	47	0.386 (0.232~0.641)		0.494 (0.281~0.867)	

## Abbreviations

PDAC: pancreatic ductal adenocarcinoma
HLEC: human lymphatic endothelial cell
EV: extracellular vesicle
MicroRNA: miRNA
TCGA: The Cancer Genome Atlas Program
GTEx: The Genotype-Tissue Expression
TMA: tissue microarray
ISH: *in situ* hybridization
ECM: Endothelial Cell Medium
qRT-PCR: Quantitative RT-PCR
SEC: Size Exclusion Chromatography
SRB: Sulforhodamine B
ELISA: Enzyme-linked immunosorbent assay
OD: optical density
NTA: nanoparticle size analysis
CAMS: Cell adhesion molecules
HPNE: normal pancreatic ductal cell line
TAM: tumor-associated macrophages

## References

[1] Rahib L, Wehner MR, Matrisian LM, Nead KT (2021). Estimated Projection of US Cancer Incidence and Death to 2040. JAMA Netw Open.

[2] Kleeff J, Korc M, Apte M, La Vecchia C, Johnson CD, Biankin AV (2016). Pancreatic cancer. Nat Rev Dis Primers.

[3] Halbrook CJ, Lyssiotis CA, Pasca di Magliano M, Maitra A (2023). Pancreatic cancer: Advances and challenges. Cell.

[4] Stacker SA, Williams SP, Karnezis T, Shayan R, Fox SB, Achen MG (2014). Lymphangiogenesis and lymphatic vessel remodelling in cancer. Nat Rev Cancer.

[5] Kalluri R, LeBleu VS (2020). The biology, function, and biomedical applications of exosomes. Science.

[6] Qin C, Zhao B, Wang Y, Li T, Li Z, Li T (2023). Exosome-mediated cell-cell communication within pancreatic cancer tumor microenvironment: a narrative review. Journal of Pancreatology.

[7] Kalluri R, McAndrews KM (2023). The role of extracellular vesicles in cancer. Cell.

[8] Casari I, Howard JA, Robless EE, Falasca M (2021). Exosomal integrins and their influence on pancreatic cancer progression and metastasis. Cancer Lett.

[9] Costa-Silva B, Aiello NM, Ocean AJ, Singh S, Zhang H, Thakur BK (2015). Pancreatic cancer exosomes initiate pre-metastatic niche formation in the liver. Nat Cell Biol.

[10] Melo SA, Luecke LB, Kahlert C, Fernandez AF, Gammon ST, Kaye J (2015). Glypican-1 identifies cancer exosomes and detects early pancreatic cancer. Nature.

[11] Li B, Cao Y, Sun M, Feng H (2021). Expression, regulation, and function of exosome-derived miRNAs in cancer progression and therapy. Faseb j.

[12] Wang X, Luo G, Zhang K, Cao J, Huang C, Jiang T (2018). Hypoxic Tumor-Derived Exosomal miR-301a Mediates M2 Macrophage Polarization via PTEN/PI3Kγ to Promote Pancreatic Cancer Metastasis. Cancer Res.

[13] Li M, Zhou J, Zhang Z, Li J, Wang F, Ma L (2022). Exosomal miR-485-3p derived from pancreatic ductal epithelial cells inhibits pancreatic cancer metastasis through targeting PAK1. Chin Med J (Engl).

[14] Nielsen BS (2012). MicroRNA *in situ* hybridization. Methods Mol Biol.

[15] Min L, Wang B, Bao H, Li X, Zhao L, Meng J, Wang S (2021). Advanced Nanotechnologies for Extracellular Vesicle-Based Liquid Biopsy. Adv Sci (Weinh).

[16] Essandoh K, Yang L, Wang X, Huang W, Qin D, Hao J (2015). Blockade of exosome generation with GW4869 dampens the sepsis-induced inflammation and cardiac dysfunction. Biochim Biophys Acta.

[17] Wang Y, Qin C, Zhao B, Li Z, Li T, Yang X (2023). EGR1 induces EMT in pancreatic cancer via a P300/SNAI2 pathway. J Transl Med.

[18] Liu T, Chen Z, Chen W, Evans R, Xu J, Reeves ME (2023). Dysregulated miRNAs modulate tumor microenvironment associated signaling networks in pancreatic ductal adenocarcinoma. Precis Clin Med.

[19] Claesson-Welsh L, Welsh M (2013). VEGFA and tumour angiogenesis. J Intern Med.

[20] Mayoral-Varo V, Calcabrini A, Sánchez-Bailón MP, Martín-Pérez J (2017). miR205 inhibits stem cell renewal in SUM159PT breast cancer cells. PLoS One.

[21] Mugisha S, Di X, Wen D, Zhao Y, Wu X, Zhang S, Jiang H (2022). Upregulated GATA3/miR205-5p Axis Inhibits MFNG Transcription and Reduces the Malignancy of Triple-Negative Breast Cancer. Cancers (Basel).

[22] Shen Y, Xu Y, Huang L, Chi Y, Meng L (2021). MiR-205 suppressed the malignant behaviors of breast cancer cells by targeting CLDN11 via modulation of the epithelial-to-mesenchymal transition. Aging (Albany NY).

[23] Kalogirou C, Linxweiler J, Schmucker P, Snaebjornsson MT, Schmitz W, Wach S (2021). MiR-205-driven downregulation of cholesterol biosynthesis through SQLE-inhibition identifies therapeutic vulnerability in aggressive prostate cancer. Nat Commun.

[24] He L, Zhu W, Chen Q, Yuan Y, Wang Y, Wang J, Wu X (2019). Ovarian cancer cell-secreted exosomal miR-205 promotes metastasis by inducing angiogenesis. Theranostics.

[25] Wei J, Zhang L, Li J, Zhu S, Tai M, Mason CW (2017). MicroRNA-205 promotes cell invasion by repressing TCF21 in human ovarian cancer. J Ovarian Res.

[26] Peach CJ, Mignone VW, Arruda MA, Alcobia DC, Hill SJ, Kilpatrick LE, Woolard J (2018). Molecular Pharmacology of VEGF-A Isoforms: Binding and Signalling at VEGFR2. Int J Mol Sci.

[27] Almalki SG, Agrawal DK (2017). ERK signaling is required for VEGF-A/VEGFR2-induced differentiation of porcine adipose-derived mesenchymal stem cells into endothelial cells. Stem Cell Res Ther.

[28] Liu X, He H, Zhang F, Hu X, Bi F, Li K (2022). m6A methylated EphA2 and VEGFA through IGF2BP2/3 regulation promotes vasculogenic mimicry in colorectal cancer via PI3K/AKT and ERK1/2 signaling. Cell Death Dis.

[29] Bui HM, Enis D, Robciuc MR, Nurmi HJ, Cohen J, Chen M (2016). Proteolytic activation defines distinct lymphangiogenic mechanisms for VEGFC and VEGFD. J Clin Invest.

[30] Hirakawa S, Kodama S, Kunstfeld R, Kajiya K, Brown LF, Detmar M (2005). VEGF-A induces tumor and sentinel lymph node lymphangiogenesis and promotes lymphatic metastasis. J Exp Med.

[31] Mandal R, Şenbabaoğlu Y, Desrichard A, Havel JJ, Dalin MG, Riaz N (2016). The head and neck cancer immune landscape and its immunotherapeutic implications. JCI Insight.

[32] Kajal K, Bose S, Panda AK, Chakraborty D, Chakraborty S, Pati S (2021). Transcriptional regulation of VEGFA expression in T-regulatory cells from breast cancer patients. Cancer Immunol Immunother.

[33] Terme M, Pernot S, Marcheteau E, Sandoval F, Benhamouda N, Colussi O (2013). VEGFA-VEGFR pathway blockade inhibits tumor-induced regulatory T-cell proliferation in colorectal cancer. Cancer Res.

[34] Lampert EJ, Zimmer A, Padget M, Cimino-Mathews A, Nair JR, Liu Y (2020). Combination of PARP Inhibitor Olaparib, and PD-L1 Inhibitor Durvalumab, in Recurrent Ovarian Cancer: a Proof-of-Concept Phase II Study. Clin Cancer Res.

[35] Incio J, Tam J, Rahbari NN, Suboj P, McManus DT, Chin SM (2016). PlGF/VEGFR-1 Signaling Promotes Macrophage Polarization and Accelerated Tumor Progression in Obesity. Clin Cancer Res.

