# Supplementary material for: Extracellular vesicles-miR-205-5p inhibits lymphatic metastasis in pancreatic cancer through diffusely downregulating VEGFA
Source: J Cancer. 2025 Mar 29;16(7):2197–211. doi: 10.7150/jca.110659 (PMC12036096; doi:10.7150/jca.110659)
Supplement: Supplementary file 1 — Supplementary figures and data legend. [file jcav16p2197s1.pdf]

Supplementary material

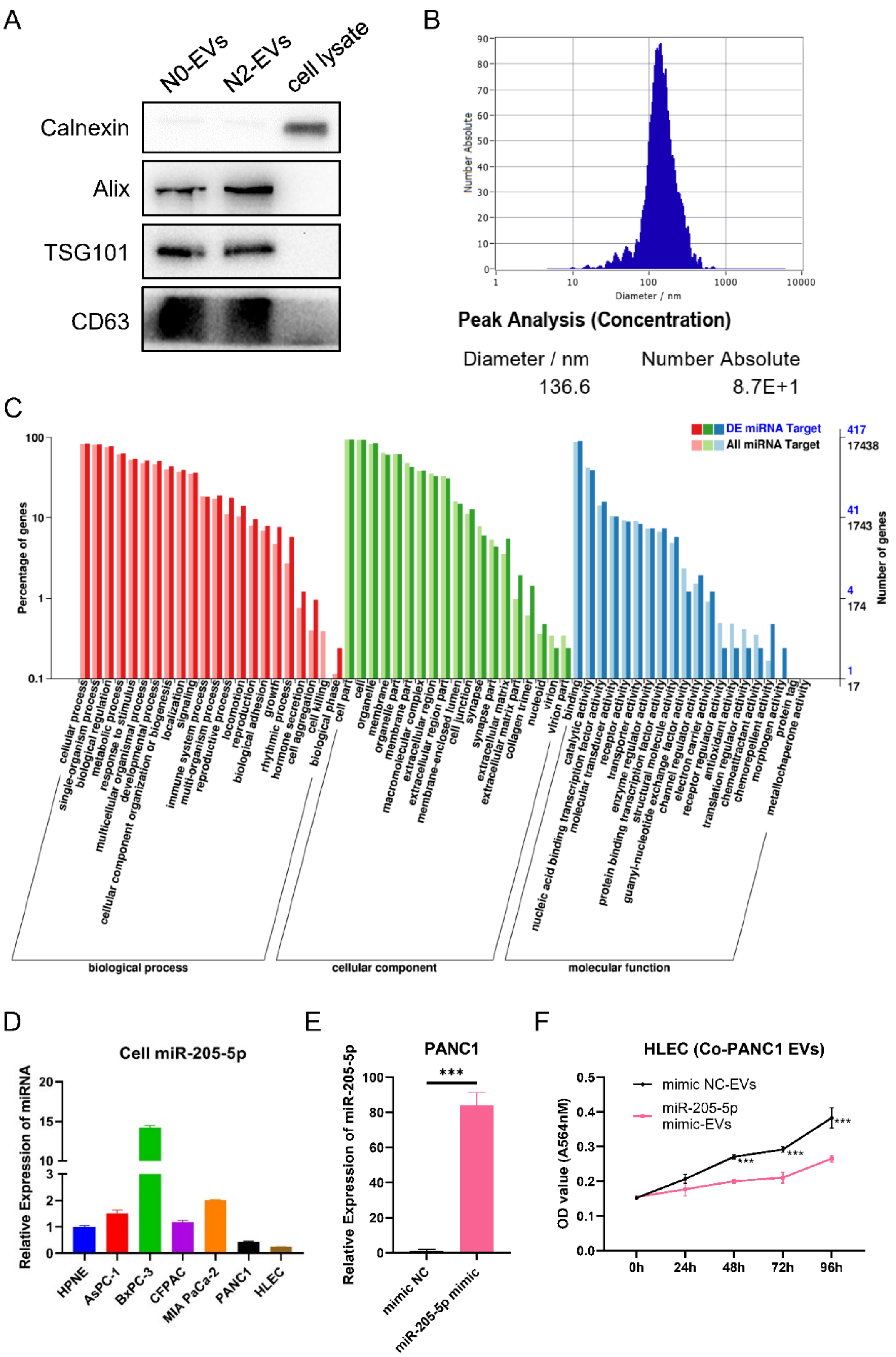

Supplementary Figure 1. (A) The positive EV-protein markers of were detected in

EVs or cell lysate. (B) The NTA result of extracted EVs. (C) GO analysis of all miRNAs-targets and differentially expressed miRNA-targets. (D) The relative mRNA expression of miR-205-5p in multiple cell lines was shown. (E) The effect of mimic miR-205-5p in PANC1 was verified by qRT-PCR ($P < 0.001$). (F) The proliferation rate of HLEC was tested after co-cultured with miR-205-5p-overexpressed PANC1-EVs. ***: $P < 0.001$.

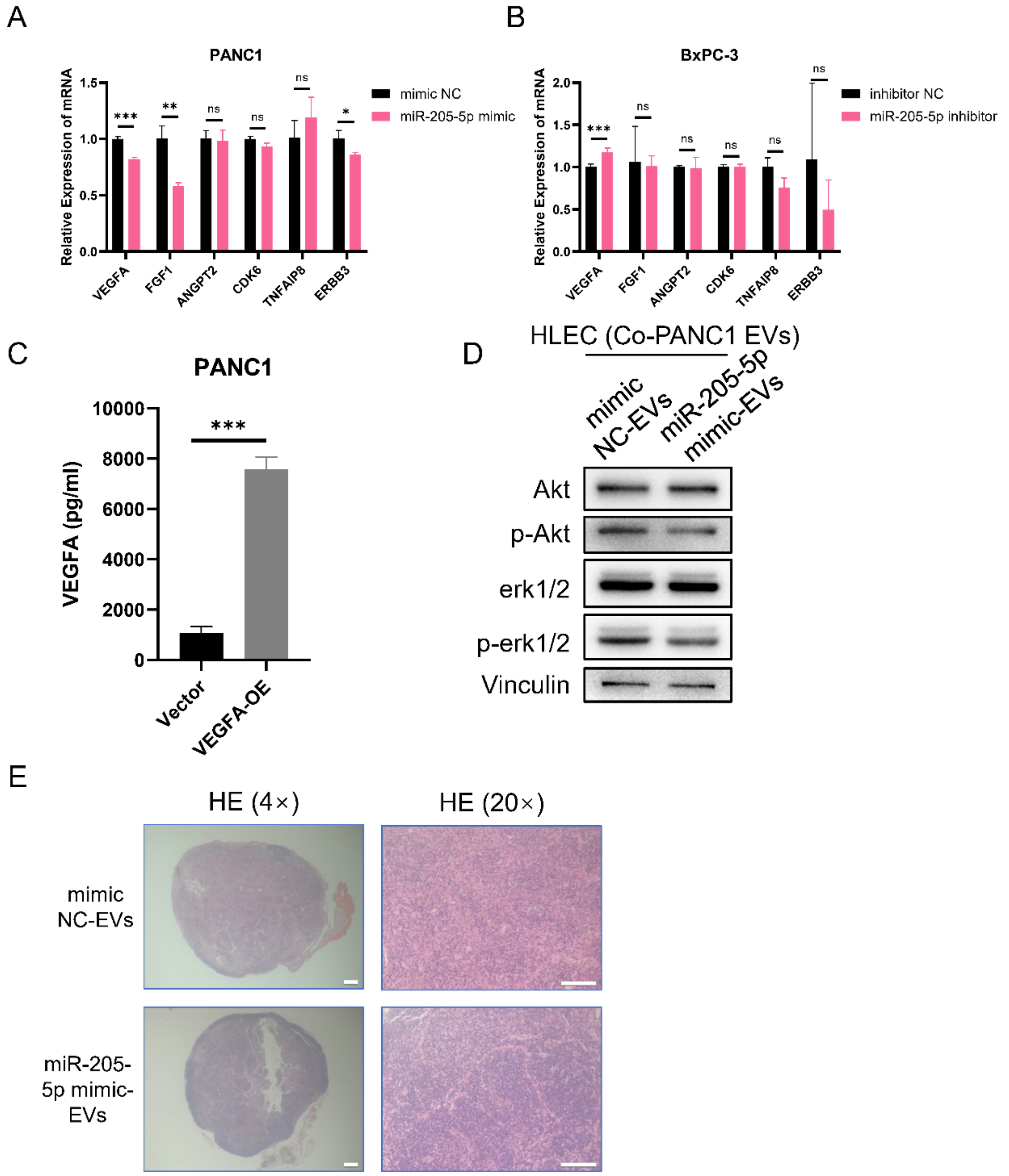

Supplementary Figure 2. (A-B) Several potential downstream genes of miR-205-5p were tested after mimic miR-205-5p in PANC1 or inhibition of miR-205-5p in BxPC-3. (C) The concentration of cell secretory VEGFA was compared after overexpression of VEGFA in PANC1. (D) The expression of VEGFA downstream molecules were tested after co-cultured with miR-205-5p-overexpressed PANC1-EVs in HLEC. (E) HE staining of the popliteal lymph nodes of PANC1 with CON-EVs or MIR205-

overexpressed EVs were shown. Scale bars left: 200 μm. Scale bars right: 100 μm. *: $P < 0.05$. **: $P < 0.01$. ***: $P < 0.001$.

Supplementary Data 1. The *P* value and fold change of the miRNAs in each sample.
